# Prevalence and clinical significance of point of care elevated lactate at emergency admission in older patients: a prospective study

**DOI:** 10.1007/s11739-022-03005-w

**Published:** 2022-06-09

**Authors:** Mélanie Gosselin, Cédric Mabire, Mathieu Pasquier, Pierre-Nicolas Carron, Olivier Hugli, Françcois-Xavier Ageron, Fabrice Dami

**Affiliations:** 1 Emergency Department, Lausanne University Hospital, University of Lausanne, 1011 Lausanne, Switzerland; 2 Institute of Higher Education and Research in Healthcare, Lausanne University Hospital and University of Lausanne, Lausanne, Switzerland

**Keywords:** Lactate, Point-of-care, Poor outcome, Emergency, NEWS, MEWS, qSOFA, Older people

## Abstract

**Objective:**

Patients who are over 65 years old represent up to 24% of emergency department (ED) admissions. They are at increased risk of under-triage due to impaired physiological responses. The primary objective of this study was to assess the prevalence of elevated lactate by point of care testing (POCT) in this population. The secondary objective was to assess the additional value of lactate level in predicting an early poor outcome, as compared to and combined with common clinical scores and triage scales.

**Methods:**

This monocentric prospective study recruited ED patients who were over 65 years old between July 19th 2019 and June 17th 2020. Patients consulting for seizures or needing immediate assessment were excluded. POCT lactates were considered elevated if ≥ 2.5 mmol/L. A poor outcome was defined based on certain complications or therapeutic decisions.

**Results:**

In total, 602 patients were included; 163 (27.1%) had elevated lactate and 44 (7.3%) had a poor outcome. There was no association between poor outcome and lactate level. Modified Early Warning Score (MEWS) was significantly associated with poor outcome, alongside National Early Warning Score (NEWS). Logistic regression also associated lactate level combined with MEWS and poor outcome.

**Conclusion:**

The prevalence of elevated lactate was 27.1%. Lactate level alone or combined with different triage scales or clinical scores such as MEWS, NEWS and qSOFA was not associated with prediction of a poor outcome. MEWS alone performed best in predicting poor outcome. The usefulness of POCT lactate measurement at triage is questionable in the population of 65 and above.

**Supplementary Information:**

The online version contains supplementary material available at 10.1007/s11739-022-03005-w.

## Introduction

### Background

Hospital-based emergency departments (ED) use triage scale systems to prioritize patients by severity and medical needs, with the first level requiring immediate assessment [1–5]. Triage is important in settings with prolonged waiting times or to identify patients with time-sensitive conditions [6]. Performed by experienced nurses, triage is used to inquire about chief complaints, comorbidities, global appearance and vital signs, sometimes using clinical scores such as the Modified Early Warning Score (MEWS) [7], National Early Warning Score (NEWS) [8] and quick Sequential Organ Failure Assessment (qSOFA) [9, 10]. Under-triage is problematic, as it delays management and care, and can impact patient outcomes.

### Importance

Patients aged 65 years and older represent up to 24% of ED admission cases [11]. These patients are at increased risk of under-triage due to their impaired physiological responses, which blunt the usual clinical signs [12–15]. In addition, the frequent combination of polymorbidity, polypharmacy, and cognitive impairment further impedes clinical assessment [16, 17]. As a result, good clinical practice standards uphold that normal vital signs do not rule out significant illness in the elderly [18]. It is therefore not surprising that the performance of the conventional triage scale has been found to be inferior in older patients [19–23] raising additional concerns about under-triage in this population.

Lactate levels have been of particular interest, based on their ability to refine prediction of patients’ prognosis in the ED [24–27] intensive care unit (ICU) [28] or prehospital settings [29, 30]. Among ED patients, higher rates of early mortality, particularly due to sepsis [31, 32] and trauma [33, 34], have been associated with high levels of lactate. Elevated lactate may also be a useful prognostic indicator in ED patients with cardiac disease, gastrointestinal disease, and bleeding [35].

Point-of-care testing (POCT) is reliable for measuring lactate levels and could be particularly useful as an easy and rapid tool for risk categorisation at ED triage [36–38] Although arterial lactate level has long been considered the reference level, several studies have showed an excellent correlation between arterial measurements and capillary POCT testing when applied in septic patients in the ICU, ED and prehospital settings [39–42].

### Goals of this investigation

The primary objective of the present study was to prospectively assess the prevalence of elevated lactate by POCT in a non-critically ill elderly population presenting to the ED for any complaint. The secondary objective was to assess the additional value of lactate level in predicting a poor outcome during or immediately after ED work up, as compared to and combined with the Swiss Emergency Triage Scale (SETS) and other validated clinical scores (MEWS, NEWS, qSOFA).

## Methods

### Study design and setting

This monocentric prospective study included patients admitted between July 19th 2019 and June 17th 2020 during opening business hours [43]. Due to the public health emergency caused by SARS-CoV-2, research resources were diverted to medical care and the recruitment was suspended from March 7th to April 26th 2020. Patients’ written consent or that from their legal or family representative was obtained.

This study took place in a Swiss ED admitting over 67,000 patients/year as part of an urban academic tertiary care university hospital with 1400 beds. This ED uses the Swiss Emergency Triage Scale (SETS), a validated 4-level triage scale. Trained ED nurses apply triage on patients’ arrival (SETS 1 category: Life/limb-threatening situation requiring immediate assessment; SETS 2 category: potentially life-threatening situation requiring assessment within 20 min; SETS 3 category: situation requiring assessment within 120 min; and SETS 4 category: non-urgent situations) [23]. For comparison, the SETS level 1 definition is similar to the Australasian scale level 1 (life-threatening condition, must be seen immediately) [44], the Emergency severity index level 1 (patient requires immediate lifesaving intervention) [3], or the Manchester triage scale (level 1: immediate care) [9, 22].

### Participants

Patients aged 65 years and older who were admitted to the ED between 8 am and 5 pm were eligible. SETS 1 patients were excluded due to their need for immediate assessment regardless of lactate level. Patients were excluded if they had already been included in this study, had a detainee status, or if they benefitted from a medical assessment within the ED prior to screening by research nurses (RN). Patients admitted for seizures were also excluded, as seizure-related increased lactate levels may not have the same prognostic value as in other illnesses or trauma [45, 46].

### Interventions

RNs saw eligible patients within 2 h following ED triage, before any medical assessment or treatment (medication, fluids, oxygen) was initiated.

In this study, the StatStrip Xpress Lactate Meter capillary device (Nova Biomedical™) was used [47]. Analysis of its performance has previously demonstrated excellent correlation and concordance with the reference laboratory methods in an ICU population [39]. The device was calibrated every 2 days following the manufacturer’s instructions. Cut-offs for elevated lactate levels are not universally agreed upon [48]. In this study, blood lactate levels were considered normal when < 2.5 mmol/L, intermediate from 2.5 to 3.9 mmol/L, and high when ≥ 4.0 mmol/L. Three lactate level groups were also used in the study by Contenti et al. [45].

### Measurements

Patient demographics, time and means of arrival, level of responsiveness, and vital signs at triage were obtained through the hospital electronic health records and completed, if needed, directly at the bedside by RNs. Data on the interval from arrival to POCT measurement, lactate levels, and supplementary O_2_ provided at triage were also collected. Information was then reported in a coded identity database (REDCap™ Vanderbilt University, Nashville, TN, USA). After obtaining written informed consent from the patients or, if necessary, from their family member at the bedside or their therapeutic representative, the RNs proceeded to take a lactate measurement. The values obtained were not relayed to the patient or ED professionals. As the study took place in an emergency setting, temporary presumptive consent could be accorded for 48 h by an ED practitioner not involved in the study. Patients without decision-making capacity on arrival could be included if written consent was obtained within 48 h of admission.

### Clinical score: MEWS, NEWS, qSOFA

The three scores used, unlike the SOFA (Sequential Organ Failure Assessment) or NEWS 2, which require laboratory assessments, were completed based on the clinical triage data. The scores were applied according to their original description: MEWS (Sup. 1) and NEWS (Sup. 2) were considered positive if the score was ≥ 5 or if any single physiological parameter was scored + 3, while qSOFA (Sup. 3) was considered positive if scored ≥ 2 points.

### Outcome measurement

Poor outcomes were defined based on previous research. [49, 50]: high levels of IV fluid supplementation (500 mL or more per 30 min), vasoactive medication (epinephrine, ephedrine, phenylephrine, noradrenaline), intubation, non-invasive ventilation or high-flow nasal oxygen therapy, thrombolysis for suspected pulmonary embolism, cardiac arrest or death during the index ED visit, or ICU or intermediate care unit admission following ED workup.

Emergent ancillary investigations or treatments such as radiology exams, coronary angiography, or surgery were not considered, as they are heavily influenced by the global patient flow.

### Analysis

Results are presented using standard descriptive statistics: proportions, means with standard deviation, or medians with interquartile range, as appropriate. The association of poor outcome with clinical scores and lactate levels was tested using a logistic regression model. To develop a nomogram that can be easily used at the bedside, continuous variables were converted to dichotomous variables using the cut-off value for each of the clinical scores (NEWS, MEWS and qSOFA). First, univariable logistic regression analyses were performed on the association between poor patient outcomes and lactate levels, as well as the dichotomised variables SETS 2, SETS 3, SETS 4, NEWS positive, MEWS positive and qSOFA positive. Clinical usefulness of lactate levels and clinical scores were evaluated using decision curve analysis (DCA), as described by Vickers et al. [51]. Then, bivariable logistic regression analyses were used to assess the association between poor outcome and lactate level combined with SETS 2 or SETS 3, SETS 4, NEWS positive, MEWS positive, or qSOFA positive status. Post-estimation analyses using the continuous scores of the predictor variables (SETS, NEWS, MEWS and qSOFA) showed similar regression coefficients as the dichotomous variables.

The classification performance of each logistic regression model was measured by calculating its area under the ROC curve. Areas under ROC curves were compared using an algorithm suggested by DeLong[52], using the roccomp command in Stata 17.0 (Statacorp, TX, USA). A two-tailed *p* value of < 0.05 was considered statistically significant.

The study was approved by the local state’s ethics committee (Req-2019-01232).

## Results

### Characteristics of study subjects

**Fig. 1:**
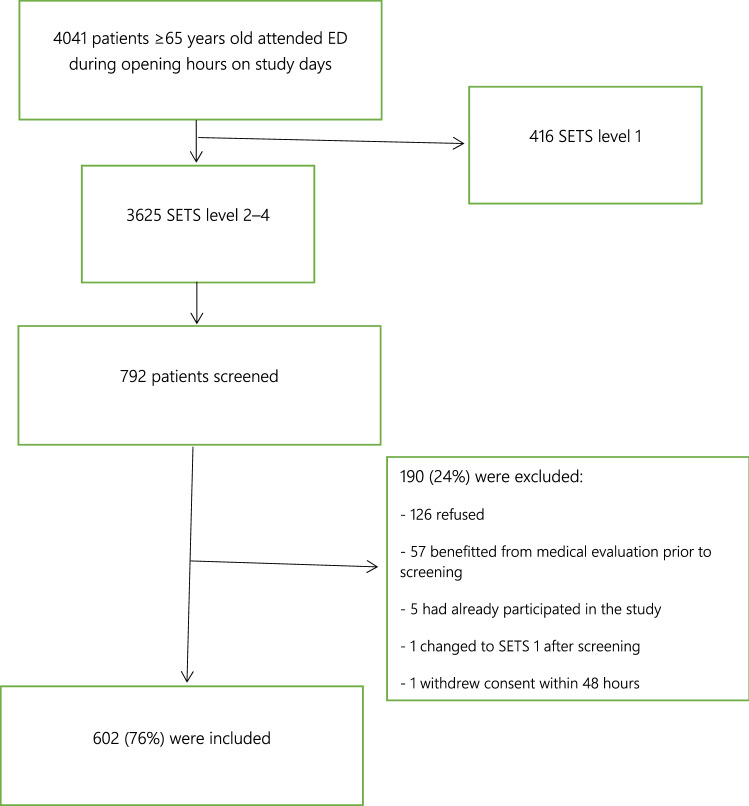
Flowchart

In total, 4041 patients over 65 years old attended the ED during opening hours on the study days. Among the 792 patients who met the inclusion criteria, 190 (24%) were excluded (126 immediately refused, 57 benefitted from a medical evaluation before an RN could perform their evaluation, five were not eligible due to having already participated in the study, one had their triage level changed to SETS 1 after initial evaluation, and one withdrew consent within 48 h of admission) (Fig. 1).

**Fig. 2:**
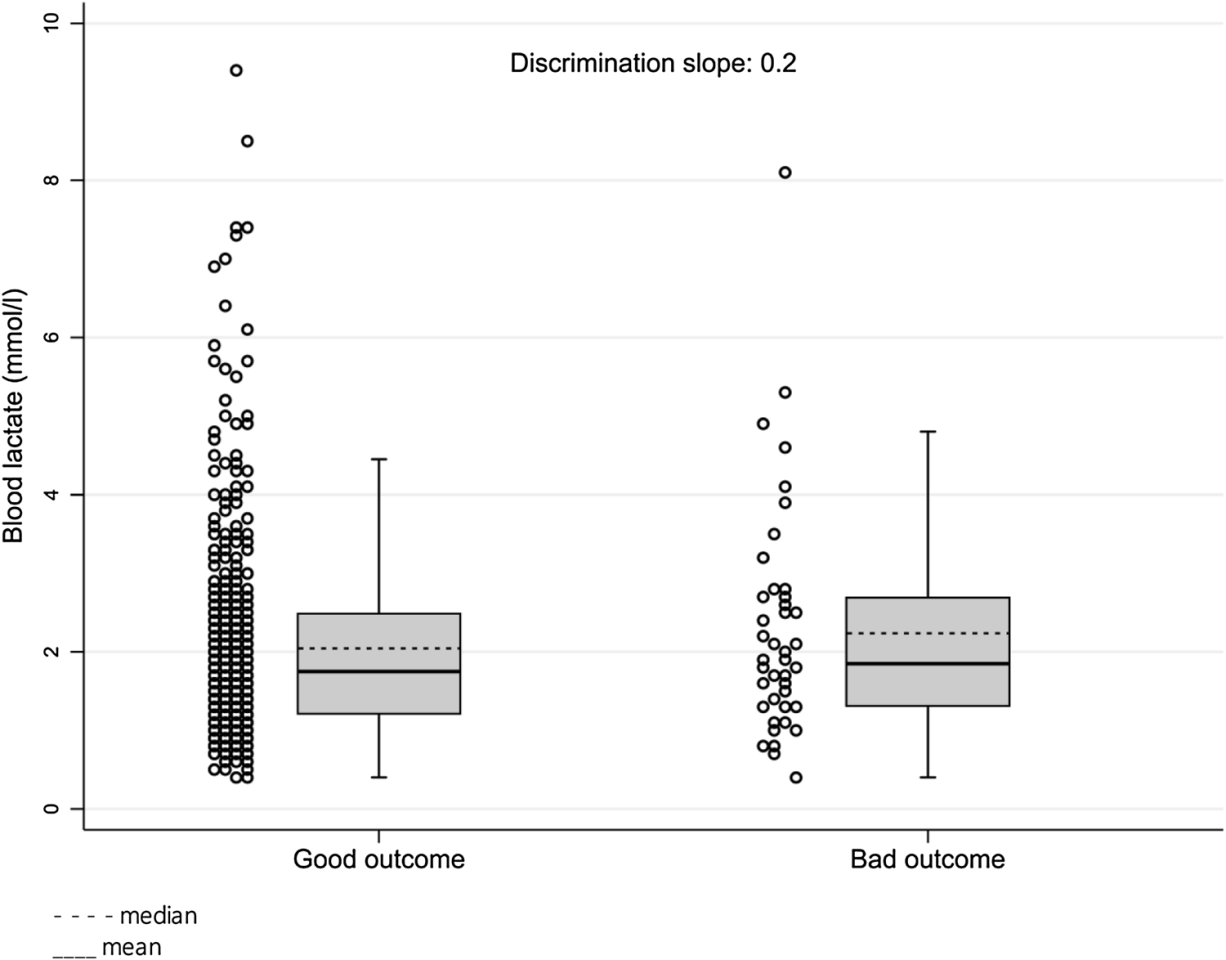
Blood lactate concentration distribution for patients with and without bad outcome (dashed line—median, line—mean)

Overall, 602 patients were included. Written consent was obtained directly from either the patient (92.5%) or their representative (7.5%). The median age was 80 years old (IQR = 12) and 44.9% (*N* = 270) were males. In total, 208 patients (34.6%) were triaged as SETS level 2, 381 (63.3%) as level 3, and 13 (2.2%) as level 4. The median capillary blood lactate level was 1.8 mmol/L, with extremes varying from 0.4 to 9.4 mmol/L (IQR = 1.3) (Table 1). Figure 2 shows the lactate level distribution for both patients with and without poor outcomes. The distribution is not normal (skewness = 2.03 and kurtosis = 9.11).

**Table 1:** Characteristics of study subjects, blood lactate levels and clinical scores

	Total (*n* = 602)	LCBL (*n* = 439)	ICBL (*n* = 121)	HCBL (*n* = 42)	ICBL + HCBL (n = 163)
Age median [years]	80	81	80	78	79
Sex male (*n*; %)	270 (44.9)	203 (46.2)	53 (43.8)	14 (33.3)	67 (41.1)
Age > 80 (*n*; %)	292 (48.5)	221 (50.3)	56 (46.3)	15 (35.7)	71 (43.6)
SETS 2 (*n*; %)	208 (34.6)	149 (33.9)	40 (33.1)	19 (45.2)	59 (36.2)
SETS 3 (*n*; %)	381 (63.3)	279 (63.6)	80 (66.1)	22 (52.4)	102 (62.6)
SETS 4 (*n*; %)	13 (2.2)	11 (2.5)	1 (0.8)	1 (2.4)	2 (1.2)
NEWS mean (SD)	2.28 (2.22)	2.30 (2.25)	2.0 (2.03)	2.89 (2.44)	2.23 (2.17)
NEWS nb positive	153 (25.4%)	112 (25.5%)	25 (20.7%)	16 (38.1%)	41 (25.2%)
MEWS mean (SD)	1.37 (1.09)	1.40 (1.1)	1.25 (1.04)	1.40 (1.19)	1.29 (1.08)
MEWS nb positive	21 (3.5%)	14 (3.2%)	5 (4.1%)	2 (4.8%)	7 (4.3%)
qSOFA mean (SD)	0.26 (0.47)	0.28 (0.49)	0.16 (0.36)	0.29 (0.51)	0.19 (0.41)
qSOFA nb positive	10 (1.7%)	9 (2.1%)	0	1 (2.4%)	1 (0.6%)
Lactate-median [mmol/L]	1.8	1.5	2.9	4.9	3.2

LCBL low capillary blood lactate (lactate < 2.5 mmol/L), *ICBL* intermediate capillary blood lactate (2.5 ≥ lactate < 4 mmol/L), *HCBL* high capillary blood lactate (lactate ≥ 4 mmol/L)

### Main results

**Fig. 3:**
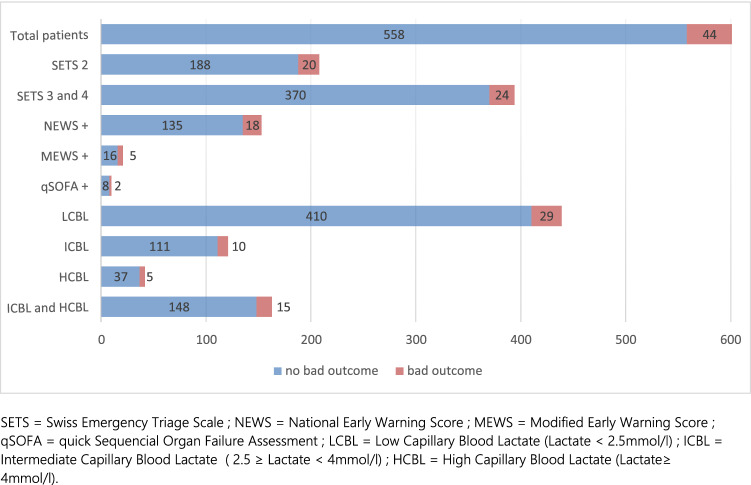
Overview of poor outcomes in different prediction tools. *SETS* Swiss Emergency Triage Scale, *NEWS* National Early Warning Score, *MEWS* Modified Early Warning Score, *qSOFA* quick Sequencial Organ Failure Assessment, *LCBL* low capillary blood lactate (lactate < 2.5 mmol/L), *ICBL* intermediate capillary blood lactate (2.5 ≥ lactate < 4 mmol/L), *HCBL* high capillary blood lactate (lactate ≥ 4 mmol/L)

According to the predefined cut-off, 163 patients (27.1%) had elevated (intermediate or high) capillary blood lactate (≥ 2.5 mmol/L).Forty-four (7.3%) of the 602 included patients had a poor outcome (Fig. 3). More specifically, 32 patients required a transfer to an intermediate care unit, two patients were transferred to the ICU, four benefited from non-invasive ventilation or high-flow nasal oxygen therapy, and nine required a high-volume IV infusion. In addition, two patients died during ED workup (one from urosepsis and one from severe respiratory failure). One patient admitted for fracture died within 48 h of ED admission, after his transfer to the orthopedic surgery ward.

**Fig. 4:**
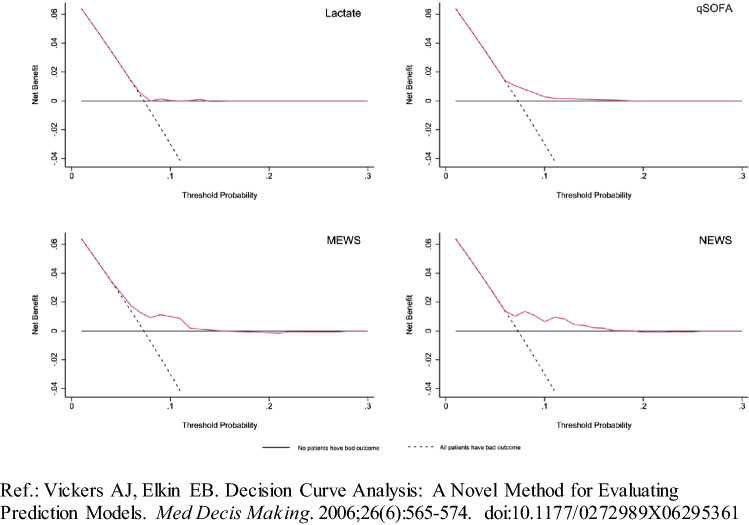
Discrimination curve representing the potential clinical usefulness of lactates and clinical scores. Ref. [51]

There was no association between poor outcome and lactate level (OR = 1.12, *p* = 0.31), SETS 2 and SETS 3 triage level (OR = 1.64, *p* = 0.12 and OR = 0.68, *p* = 0.21, respectively). In contrast, MEWS score was significantly associated with poor outcome (OR = 4.34; *p* < 0.01; AUC = 0.54) as well as NEWS (OR = 2.17; *p* < 0.02; AUC = 0.58). qSOFA score was not significantly associated with poor outcome (OR = 3.27; *p* = 0.14) (Table 2; Fig. 4).

**Table 2:** Univariate and bivariate logistic regression model to predict poor outcome

Poor outcome	Univariate model			Bivariate model			
	OR	95% CI	AUC		OR	95% CI	AUC
Lactate	1.12	[0.90; 1.40]	0.53	Lactate	1.12	[0.89; 1.39]	0.58
SETS 2	1.64	[0.88; 3.05]	0.56	SETS2	1.63	[0.88; 3.02]	
SETS 3	0.68	[0.36; 1.25]	0.54	Lactate	1.12	[0.90; 1.39]	0.56
SETS 4	0.08	[0.06; 0.11]	0.59	SETS3	0.68	[0.37; 1.26]	
MEWS positive	4.34*	[1.51; 12.48]	0.54	Lactate	1.12	[0.90; 1.39]	0.53
NEWS positive	2.17*	[1.15; 4.08]	0.58	SETS4	–	–	
qSOFA positive	3.27	[0.67; 15.91]	0.52	Lactate	1.10	[0.88; 1.37]	0.58
				MEWS	4.17*	[1.44; 12.06]	
				Lactate	1.11	[0.89; 1.38]	0.60
				NEWS	2.15*	[1.14; 4.05]	
				Lactate	1.11	[0.89; 1.39]	0.54
				qSOFA	3.20	[0.65; 15.67]	
				SETS2	1.55	[0.83; 2.90]	0.58
				MEWS	4.0*	[1.39; 11.68]	
				SETS2	1.51	[0.81; 2.83]	0.60
				NEWS	2.1*	[1.09; 3.90]	
				SETS2	1.56	[0.83; 2.93]	0.56
				qSOFA	2.69	[0.54; 13.40]	

*AUC* area under ROC curve, *CI* confidence interval, *OR* odds ratio

*p < 0.05

Logistic regression was applied in an attempt to predict poor outcome. The combination of lactate level and MEWS significantly predicted poor outcome (OR = 4.17; *p* < 0.01; AUC = 0.58), but with a lower OR than MEWS alone. Lactate level combined with NEWS score (OR = 2.15; *p* < 0.02; AUC = 0.60) presented a similar OR and *p* value to NEWS alone. The combination of lactate levels with patients triaged as SETS 2 category or qSOFA was not significantly predictive of a poor outcome (OR = 1.63; *p* = 0.123; OR = 3.20; *p* = 0.152, respectively) (Table 2).

None of the patients who died during ED work or within 48 h had a lactate level ≥ 2.5 mmol/L.

## Limitations

We acknowledge that our study has several limitations. First, our assessment of poor outcomes was based on previous works, in the absence of consensus among ED physicians, and thus is subject to discussion [49, 50]; we cannot exclude misclassification of outcomes. Second, there may have been errors of measurement. The use of a single measurement for prognostic factors may lead to errors and regression dilution bias. Third, the relative homogeneity of the cases included in the study may have reduced the discrimination of the areas under the curves. Fourth, a convenience sample was used. Although we included consecutive patients in the presence of the RNs, an inclusion bias cannot be excluded. Fifth, recruitment had to be temporally placed on hold during the first wave of the COVID-19 pandemic, which contributed to the small number of patients included. A study with a larger population is needed to confirm our findings, as a lower than expected number of patients may have led our study to be underpowered and unable to detect a potential impact of lactate level alone. Finally, our study is monocentric, and our findings must be replicated in ED settings with a different case mix.

## Discussion

To our knowledge, this is the first prospective study to test the prognostic value of systematic capillary lactate measurements by POCT at ED triage among patients over 65 years old presenting to an urban hospital and without life-threatening conditions identified at triage. We found that the prevalence of elevated lactate levels (≥ 2.5 mmol/L) was 27.1% in this setting, and that lactate level, alone or combined with the SETS 2 category or positive qSOFA, was not associated with poor outcome. Positive NEWS or MEWS scores were associated with poor outcome, but this prediction was not improved by combining it with lactate level.

Lactate levels have been a source of interest as a prognostic biomarker in emergency medicine patients for some time. Lactate levels measured during ED workup have, in other larger studies, shown a good predictive value for 10- to 60-day mortality [24–27]. Several differences may explain the discrepancy between these results and those of our study.

First, most retrospective studies and studies selecting specific symptoms or pathologies such as sepsis or trauma are confounded by an indication bias: only the most severe patients had their lactate levels measured. The much higher prevalence of previously published high lactate levels, as high as 48% [27], may reflect this selection bias. As a result, these studies have led to inflated reported lactate level values.

Second, our outcome was not mortality, but poor outcome during or soon after ED workup. SETS 1 patients were excluded, as the information provided by lactate levels would have been marginal. Mortality is highly correlated with the clinical severity at presentation [Error! Bookmark not defined]. Instead, we decided to concentrate on patients that could potentially be under-triaged. Less than 1% of patients died during ED admission in our study.

Third, the prediction of an outcome 10 to 60 days after the initial lactate measurement may be affected by factors beyond ED interventions, and thus not be useful in guiding the level of monitoring and management in the ED.

MEWS or NEWS predict admission and in-hospital mortality in patients over 65 years old at ED admission [53]. While combining lactate measurements with NEWS or MEWS did not improve prediction of poor outcome in our study, this combination demonstrated a better predictive performance of 48-h mortality in a population of severely ill patients that needed prehospital acute life support in a previous prospective study [54]. The same result was found in another retrospective study of patients of all degrees of severity arriving at the ED with lactate level measurement [49]. In these two studies, the patients were not limited to those that were 65 years and older and lactates measurement were performed on selected patients.

SETS performed poorly for predicting poor outcome in this elderly population. A previous study on the Manchester Triage System (MTS) also found poor performance in the prediction of in-hospital mortality [19].

As mentioned above, the short-term indicator “poor outcome during or following ED workup” was specifically investigated by this study. Such an indicator could enable ED physicians to identify, at triage, patients at risk of poor outcome during their ED stay. There is, however, a lack of literature on this specific short-term indicator, which is the reason why this study was conducted. As any list of items predicting a poor outcome may be subject to criticism, a consensus should be met among ED physicians to be then able to perform some benchmarking.

Finally, since the usual criteria, such as vital signs and the current and past medical history, are less reliable among older patients, it was hypothesized that measurement of elevated lactate at triage would improve early detection of patients expected to have a poor outcome, a hypothesis that was not supported by this work. The use of lactate levels to guide orientation within the ED or level of care is questionable. MEWS was the best tool for predicting a poor outcome within the ED in this work, but this score does not include typical presenting complaints such as chest pain or acute vertigo, which may contribute to the correct assessment of short-term risk at triage. Therefore, clinical scores should not replace triage scales, but may be combined with them.

## Conclusion

The prevalence of elevated lactate was 27.1%. Lactate level alone or combined with different triage scales or clinical scores such as MEWS, NEWS and qSOFA was not associated with prediction of a poor outcome. MEWS alone performed best in predicting poor outcome. The usefulness of POCT lactate measurement at triage is questionable in the population of 65 and above.

## Supplementary Information

Below is the link to the electronic supplementary material.

Supplementary file1 (DOCX 14 KB)

Supplementary file2 (DOCX 16 KB)

Supplementary file3 (DOCX 15 KB)

## Data Availability

The datasets used and/or analyzed during the current study are available from the corresponding author on reasonable request.
