# Supplementary material for: Prevalence and clinical significance of point of care elevated lactate at emergency admission in older patients: a prospective study
Source: Intern Emerg Med. 2022 Jun 9;17(6):1803–12. doi: 10.1007/s11739-022-03005-w (PMC9178320; doi:10.1007/s11739-022-03005-w)
Supplement: Supplementary file 1 — Supplementary file1 (DOCX 14 KB) [file 11739_2022_3005_MOESM1_ESM.docx]

## Supplementary file 3 : Sequential Organ Failure Assessment (Quick) qSOFA

| **Assessment** | **Score** |
| --- | --- |
| Low blood pressure (SBP≤100 mmHg) | 1 |
| High respiratory rate (≥22 bpm) | 1 |
| Glasgow Coma Scale ≤14 | 1 |

1.

Ref.: Raith EP, Udy AA, Bailey M, et al. Prognostic Accuracy of the SOFA Score, SIRS Criteria, and qSOFA Score for In-Hospital Mortality Among Adults With Suspected Infection Admitted to the Intensive Care Unit. *JAMA*. 2017;317(3):290. doi:10.1001/jama.2016.20328
