# Supplementary material for: Prevalence and clinical significance of point of care elevated lactate at emergency admission in older patients: a prospective study
Source: Intern Emerg Med. 2022 Jun 9;17(6):1803–12. doi: 10.1007/s11739-022-03005-w (PMC9178320; doi:10.1007/s11739-022-03005-w)
Supplement: Supplementary file 2 — Supplementary file2 (DOCX 16 KB) [file 11739_2022_3005_MOESM2_ESM.docx]

## Supplementary file 1 : Modified Early Warning Score (MEWS)

| **Points** | Respiratory rate (bpm) | Oxygen saturation (%) | Temperature (C) | Systolic blood pressure (mmHg) | Pulse rate (bpm) | AVPU |
| --- | --- | --- | --- | --- | --- | --- |
| **3** |  | ≤91 |  | ≤70 |  |  |
| **2** | <9 | 92-93 | <35.0 | 71-80 | ≤40 |  |
| **1** |  | 94-95 |  | 81-100 | 41-50 |  |
| **0** | 9-14 | ≥96 | 35.0-38.4 | 101-199 | 51-100 | A |
| **1** | 15-20 |  |  |  | 101-110 | V |
| **2** | 21-29 |  | ≥ 38.5 | ≥ 220 | 111-129 | P |
| **3** | ≥30 |  |  |  | ≥130 | U |

Note : bpm breaths or beats per minute, AVPU ; level of consciousness Alert, Voice, Pain, Unresponsive

1.

Ref.: Subbe CP. Validation of a modified Early Warning Score in medical admissions. *QJM*. 2001;94(10):521-526. doi:10.1093/qjmed/94.10.521
