# Supplementary material for: Prevalence and clinical significance of point of care elevated lactate at emergency admission in older patients: a prospective study
Source: Intern Emerg Med. 2022 Jun 9;17(6):1803–12. doi: 10.1007/s11739-022-03005-w (PMC9178320; doi:10.1007/s11739-022-03005-w)
Supplement: Supplementary file 3 — Supplementary file3 (DOCX 15 KB) [file 11739_2022_3005_MOESM3_ESM.docx]

## Supplementary file 2 : National Early Warning Score (NEWS)

| **Points** | Respiratory rate (bpm) | Oxygen saturation (%) | Oxygen supplement | Temperature (C) | Systolic blood pressure (mmHg) | Pulse rate (bpm) | AVPU |
| --- | --- | --- | --- | --- | --- | --- | --- |
| **3** | ≤8 | ≤91 |  | ≤35.0 | ≤90 | ≤40 |  |
| **2** |  | 92-93 | Yes |  | 91-100 |  |  |
| **1** | 9-11 | 94-95 |  | 35.1-36.0 | 101-110 | 41-50 |  |
| **0** | 12-20 | ≥96 | No | 36.1-38.0 | 111-219 | 51-90 | A |
| **1** |  |  |  | 38.1-39.0 |  | 91-110 |  |
| **2** | 21-24 |  |  | ≥ 39.1 |  | 111-130 |  |
| **3** | ≥25 |  |  |  | ≥ 220 | ≥131 | V, P or U |

Note : bpm breaths or beats per minute, AVPU ; level of consciousness Alert, Voice, Pain, Unresponsive

1.

Ref.: Smith GB, Prytherch DR, Meredith P, Schmidt PE, Featherstone PI. The ability of the National Early Warning Score (NEWS) to discriminate patients at risk of early cardiac arrest, unanticipated intensive care unit admission, and death. *Resuscitation*. 2013;84(4):465-470. doi:10.1016/j.resuscitation.2012.12.016
